# Effect of the Nordmøre grid bar spacing on size selectivity, catch efficiency and bycatch of the Barents Sea Northern shrimp fishery

**DOI:** 10.1371/journal.pone.0277788

**Published:** 2022-12-27

**Authors:** Roger B. Larsen, Bent Herrmann, Manu Sistiaga, Jesse Brinkhof, Kristine Cerbule, Eduardo Grimaldo, Mark J. M. Lomeli

**Affiliations:** 1 UiT The Arctic University of Norway, Breivika, Tromsø, Norway; 2 SINTEF Ocean, Trondheim, Norway; 3 DTU Aqua, Technical University of Denmark, Hirtshals, Denmark; 4 Institute of Marine Research, Bergen, Norway; 5 Pacific States Marine Fisheries Commission, Newport, OR, United States of America; COISPA Tecnologia & Ricerca - Stazione Sperimentale per lo Studio delle Risorse del Mare, ITALY

## Abstract

The introduction of the Nordmøre grid in shrimp trawls has reduced the bycatch of non-target species. In the Norwegian Northern shrimp (*Pandalus borealis*) fishery, the mandatory selective gear consists of a Nordmøre grid with 19 mm bar spacing combined with a 35 mm mesh size diamond mesh codend. However, fish bycatch in shrimp trawls remains a challenge and further modifications of the gear that can improve selectivity are still sought. Therefore, this study estimated and compared the size selectivity of Nordmøre grids with bar spacings of 17 and 21 mm. Further, the effect of applying these two grids on trawl size selectivity was predicted and compared to the legislated gear configuration. Experimental fishing trials were conducted in the Barents Sea where the bottom trawl fleet targets Northern shrimp. Results were obtained for the target species and two by-catch species: cod (*Gadus morhua*) and American plaice (*Hippoglossoides platessoides*). This study demonstrated that reducing bar spacing can significantly reduce fish bycatch while only marginally affecting catch efficiency of Northern shrimp. This is a potentially important finding from a management perspective that could be applicable to other shrimp fisheries where flexibility in the use of different grid bar spacings may be beneficial to maximize the reduction of unwanted bycatch while minimizing the loss of target species.

## 1. Introduction

Northern shrimp (*Pandalus borealis*) is a commercially important species in bottom trawl fisheries in the North Atlantic and the Barents Sea [1]. This species has gained more interest over the last decade as a result of increased demand, increased product prices and a recommended annual quota of 140.000 tons in the Northeast Arctic [2]. Due to the small meshes used in the trawl body and codends, Northern shrimp fisheries have struggled for many years with large quantities of fish bycatch [3], which implied additional sorting labour onboard and an undesired impact on the stocks of commercially and ecologically important fish species.

**Fig 1:**
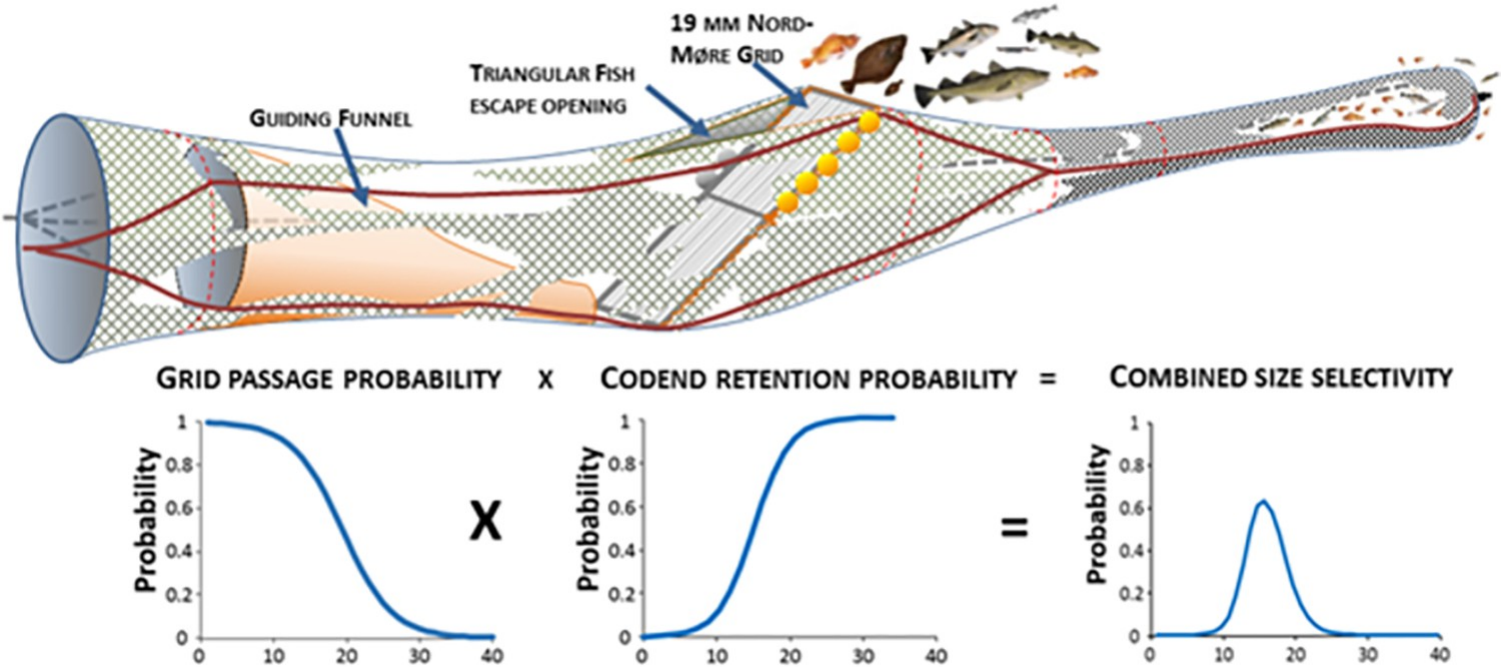
Illustration of the working principle of a sorting system built from a Nordmøre grid and a subsequent selective codend with curves representing the corresponding size dependent grid passage probability, codend retention probability and the combined size selectivity.

For most Northern shrimp fisheries, the introduction of the Nordmøre grid in the early 1990’s was a breakthrough regarding species selectivity and bycatch reduction (e.g., [4–8]). The working principle of the Nordmøre grid as legislated for the Norwegian and Russian areas of the Northeast Atlantic is shown in Fig 1. Except for a few minor fisheries in the north Atlantic where the use of the Nordmøre grid is not compulsory (e.g., fisheries in the fjords of Iceland [9], the selectivity in the majority of trawl fisheries for Northern shrimp is based upon a dual sorting system consisting of a sorting grid combined with a size selective codend. In this complex size selection process, the combined size-dependent selectivity curves for both Northern shrimp and the bycatch species often exhibit a bell-shaped signature [10] (Fig 1). This is the case for the Northern shrimp fishery in the Barents Sea. This is one of the largest fisheries for this species with annual landings of ~20.000 to ~80.000 tons in the last 20 years [2], where the use of a 19 mm bar spacing Nordmøre grid combined with a codend with a minimum mesh size of 35 mm is required [11].

The sorting grid is installed in the extension piece adjacent to the codend at an angle of approximately 45°, and a guiding funnel (or guiding panel) directs shrimp and fish towards the lower part of the grid section. The sorting system allows all organisms that pass between the bars of the grid to continue towards the codend, while those that move along the grid or cannot pass between the bars, are led out of the trawl through an escape opening in the upper panel. Consequently, larger-sized fish and other marine organisms (e.g., jellyfish, crabs, etc.) that are unable to pass through the specific grid bar spacing, are removed from the trawl making the shrimp catches in the fishery cleaner [12].

However, despite the success of the grid removing larger-sized individuals, the device does not solve the issue to the same extent for the smaller-sized individuals, especially those of sizes that are similar to shrimp which can pass between the grid bars. Thus, the management authorities of Norway and the industry are still searching for technical solutions that can further improve the performance of the Nordmøre grid.

In the Barents Sea Northern shrimp fishery, the fish bycatch such as cod (*Gadus morhua*) below minimum landing size (MLS) and American plaice (*Hippoglossoides platessoides*) has remained a problem because a fraction of small individuals that pass through the grid (i.e., up to a certain size depending on the bar spacing used in the grid) are too large to escape through the codend meshes. The regulations in the Barents Sea and all other shrimp fishing grounds under Norwegian jurisdiction regarding bycatch of specimens from quota regulated species below MLS are strict and vary between species [11]. Bycatches are controlled by the authorities during routine inspections at sea. If the catch of at least one quota regulated (i.e. commercially important) species (i.e., cod, haddock (*Melanogrammus aeglefinus*), Greenland halibut (*Reinhardtius hippoglossoides*) and two redfish species (*Sebastes* spp.)) exceeds a species-specific number of individuals per 10 kg of shrimp or more than 15% by numbers for shrimp below MLS (< 15 mm carapace length (CL)) [1], the fishing area is closed by the authorities for periods that can last weeks or months. From five defined choke species in the Barents Sea Northern shrimp fishery [11], the recruitment for cod is of great value. Cod is the most important commercial species in the Barents Sea [13–15]. The allowed bycatch amounts are determined based on the biological status of cod, as well as economic considerations concerning both the shrimp fishery and fishery for cod [13]. Specifically, when the catches of cod below MLS in this area exceed eight individuals per 10 kg of shrimp, the fishing area is closed [11]. Area closures can imply significant operational challenges and increased costs for the fishing fleet (i.e., loss of income due to access limitations to good fishing areas), and larger distances between the potential fishing grounds. Therefore, efficient selectivity measures that reduce the bycatch of species like cod are still sought. Unlike cod, American plaice is not a regulated species in the Barents Sea and, therefore, it is not subject to the same regulations. However, it is an important bycatch species to consider because it is caught in great numbers and then discarded by the fleet, which contributes to the negative ecological impact of the fishery. In addition, due to its morphology, it can block large areas of the grid reducing its sorting capacity and can cause practical problems with sorting the catch on board [15, 16].

Due to increased focus on discard reduction in fisheries [17], the efforts to improve both species and size selectivity in Northern shrimp fisheries in general (e.g., [9, 18–20]) and in the Barents Sea in particular (e.g., [21–23]) have incremented in the last decade. Many of these studies have looked on changes in the grid section design that could potentially lead to better selective properties of the grid (e.g., [24, 25]). Changing bar spacing in the Nordmøre grid is an obvious modification to change the selection characteristics of the grid section because it potentially affects the sizes of fish and Northern shrimp that can pass between the bars of the grid. However, no published study has evaluated if or how the selection properties in the Northern shrimp fishery in the Barents Sea change by modifying the Nordmøre grid bar spacing, except a brief notice of initial experiments during 1989–1990 with various bar distances in the grid [4] with few studies examining the effect of grid bar spacing in other fisheries [18, 26–29].

Thus, the aim of the present study was to investigate the effect of modifying grid bar spacing on the selectivity and catch size distribution of Northern shrimp and two relevant bycatch species in the Barents Sea Northern shrimp fishery: cod and American plaice.

Specifically, the present study aimed at answering the following research questions:

- To what extent does grid passage probability change for Northern shrimp, cod and American plaice by changing bar spacing in the Nordmøre grid?
- Is it possible to reduce the retention of cod and American plaice without changing the catch size distribution for Northern shrimp by modifying the grid bar spacing?
- What are the potential implications of changing bar spacing in the compulsory grid and codend configuration in the Barents Sea Northern shrimp fishery?

## 2. Materials and methods

### 2.1. Ethics statement

This study did not involve endangered or protected species. Experimental fishing was done on board a research vessel in accordance with the fishing permit granted by the Norwegian Directorate of Fisheries (18/18249). This fishing permit allows catches of shrimp and fish to be landed. No other permit was required to conduct this study.

### 2.2 Experimental design

The fishing trials were performed on board the research vessel "*Helmer Hanssen*" (63.8 m LOA and 4.080 HP) during 5–17 January 2019. The fishing grounds chosen for the tests were located on the West Coast of Svalbard, in Isfjorden (77°57’25”– 78°25’41” N / 11°11’28”– 15°54’46” E). The trials were conducted using a two-bridle Campelen #1800 trawl built from 3 mm polyethylene (PE) twine with 80–40 mm diamond meshes size in the wing sections and by polyamide (PA nr. 24–32) in the belly and codend sections. The trawl was towed behind a set of Thyborön T2 otter boards (6.5 m^2^ and 2.200 kg). The design used a 40 m bridle-sweep configuration and along the fishing line of the net a 19.2 m long rockhopper-type footrope with 46 cm rubber discs in the bosom and wing sections. To ensure a controlled distance between the doors of 48–52 m, independent of fishing depth and towing speed, a 20 m long restrictor rope was linked between the warps 80 m in front of the trawl doors. The height of the trawl ranged between 4.5 and 4.8 m at a towing speed of 3.0–3.2 knots (5.5–5.9 km/h). This towing speed is commonly used in the Barents Sea single, double and triple gear configurations for shrimp trawling.

Two experimental Nordmøre grids were tested, one with 16.75 ± 0.66 mm (mean ± SD) bar spacing and the other one with 20.65±0.61 mm bar spacing. The mean bar spacing of each of the grids was measured using calipers. Both grid sections were four-panel constructions in 40 mm mesh of 2.5 mm PE twine and were equivalent in circumference and length to the two-panel standard Nordmøre grid section used by the Norwegian coastal and inshore fleets targeting Northern shrimp. The Nordmøre grids were made of stainless steel and were 1510 mm high and 1330 mm wide. The grids in both sections were mounted so that they would maintain an angle of 45 ± 2.5° while fishing. The fishing trials were carried out with a selection system composed by a Nordmøre grid followed by a codend blinded with a 10 mm mesh size liner. The escape outlet on the top panel of the grid section was cut as a 1.5 m long and 1.33 m wide triangle. A small-meshed (18.9 ± 1.2 mm) cover was mounted over the outlet in front of the grid for the collection of the escaping fish and shrimp.

During the sea trials, 11 and 21 hauls were conducted with the Nordmøre grid with 17 and 21 mm bar spacing, respectively. After each tow, the catches in the codend and the cover were kept separated. All fish caught in each compartment were sorted by species and all individuals under 40 cm in total length were measured to the nearest cm below. For the fish species no subsampling was performed. However, the shrimp in each compartment were subsampled by taking a random sample of approximately 1 kg. The carapace length (CL) of all shrimp in the subsample were measured to the nearest mm below using a caliper.

### 2.3 Grid passage probability

For a shrimp or fish to pass through the grid, two conditions need to be fulfilled: 1) it needs to contact the grid; and 2) it needs to be morphologically able to pass through the grid. Thus, the escape probability for each individual will depend on their size and orientation when they contact the grid and their swimming performance and capability of avoiding the grid face. It must be considered that some shrimp and fish may not contact the Nordmøre grid at all, or that they do so with such a poor orientation that they will not have any length-dependent chance of passing through the grid. For an individual contacting the grid with sufficiently good orientation for a length-dependent probability of passing through the grid (*pc*(*l*)), the following *logit* model was used [30]:

$$pc(l,{L50}_{grid},{SR}_{grid})=1.0-\frac{exp(\frac{ln(9)}{{SR}_{grid}}\times (l-{L50}_{grid}))}{1.0+exp(\frac{ln(9)}{{SR}_{grid}}\times (l-{L50}_{grid}))}=\frac{1.0}{1.0+exp(\frac{ln(9)}{{SR}_{grid}}\times (l-{L50}_{grid}))}$$ (1)

Model 1 considers that the probability for an individual to be able to pass through the grid, under the condition that it contacts the grid, is length-dependent and decreases for larger individuals. Parameter *l* denotes the length of the individual, *L50*_*grid*_ denotes the length with a 50% probability of being prevented from passing through the grid, and the selection range (*SR*_*grid*_) describes the difference in length between individuals with a 75% and 25% probability of being prevented from passing through the grid.

Based on the above, model 2 was used for the size-dependent probability of a shrimp or fish to pass through the Nordmøre grid and enter the codend (*p*(*l*)):

$$p(l,C_{grid},{L50}_{grid},{SR}_{grid})=\frac{C_{grid}}{1.0+exp(\frac{ln(9)}{{SR}_{grid}}\times (l-{L50}_{grid}))}$$ (2)

Three parameters need to be estimated to be able to describe the size selection in the Nordmøre grid, *C*_*grid*_, *L50*_*grid*_, and *SR*_*grid*_. Parameter *C*_*grid*_, which is known as selectivity contact [31], loosely models the contact probability with the grid for modes of orientation that result in a length-dependent probability for an individual to pass through the grid. If all individuals contact the grid with a reasonable mode of contact, then the value for *C*_*grid*_ should be 1.0. However, this is not necessarily the case, and some individuals may even escape through the escape exit of the Nordmøre grid section (Fig 1) without contacting the Nordmøre grid first. Other individuals may be so poorly orientated when they meet the grid that the probability of them passing through will be similar to those not contacting the grid at all. This means that they would not have made selectivity contact [31], which will also be reflected in th e value of *C*_*grid*_. For the shrimp or fish that contact the grid with a reasonable mode of orientation, *L50*_*grid*_ and *SR*_*grid*_ describe the probability of their passage through the grid based on model 1. Shifting to larger bar spacing will result in higher *L50*_*grid*_ and altered *SR*_*grid*_ values. For small individuals that would pass through if they contacted the grid, the value 1 *–C*_*grid*_ can loosely be interpreted as the fraction of individuals that would be able to seek and subsequently escape through the escape outlet without contacting the grid. A small *SR*_*grid*_ value would indicate a well-defined grid contact orientation with all those individuals making contact doing it with a similar orientation. In contrast, a large *SR*_*grid*_ value would indicate that the contact is more disordered with many different orientations involved. As different species have different morphologies and behaviors, values of the parameters *C*_*grid*_, *L50*_*grid*_, and *SR*_*grid*_ will, for the same selective system, be species specific. Therefore, the analysis needs to be applied separately for Northern shrimp and bycatch species.

Since the aim of our study was to investigate how each of the tested Nordmøre grid configurations performed on average over the hauls conducted, the analysis included data summed over hauls *j*. The analyses were conducted separately for each Nordmøre grid configuration based on the data from the hauls with each specific grid and separately for each species. Therefore, expression (3) was minimized, which is equivalent to maximizing the likelihood for the observed data in form of the length-dependent number of individuals retained in the codend (*nC*_*jl*_) versus those collected in the Nordmøre grid cover (*nGC*_*jl*_):

$$-\sum_{j=1}^{m}\sum_{l}\left\{\frac{{nC}_{jl}}{{qC}_{j}}\times ln(p(l,C_{grid},{L50}_{grid},SR_{grid}))+\frac{{nGC}_{jl}}{{qGC}_{j}}\times ln(1.0-p(l,C_{grid},{L50}_{grid},SR_{grid}))\right\},$$ (3)

where *qC*_*j*_ and *qGC*_*j*_ are the sampling factors for the fraction of individuals measured in the catches from codend and grid cover, respectively. The sampling factors comprise a value in the range 0.0 to 1.0, with 1.0 indicating all individuals being length measured. The outer summation in expression (3) comprises the hauls conducted with the specific Nordmøre grid configuration and the inner summation over length classes in the data.

Evaluating the ability of model (2) to describe the data sufficiently well was based on calculating the corresponding *p*-value. In case of poor fit statistics (*p*-value < 0.05), the residuals were inspected to determine whether the poor result was due to structural problems when modelling the experimental data (model 2), or due to over-dispersion in the data [32].

To account for both within- and between-haul variations in selectivity [33] when estimating the uncertainty for the average size dependent grid passage probability (model 2), we applied a double bootstrap method using the software tool SELNET [34]. For each species analyzed, 1000 bootstrap repetitions were conducted to estimate the 95% confidence intervals (CI’s) (Efron percentile [35]) for the model parameters (*C*_*grid*_, *L50*_*grid*_, and *SR*_*grid*_) [34].

The effect on grid passage probability by changing grid bar spacing from *x* to *y* was evaluated based on plotting the estimated grid passage probability curve with CI’s for each of these configurations against the equivalent baseline configuration curve for the 19 mm bar spacing design applied in this fishery since 1991 [12]. Further, the difference in the length-dependent passage probability (Δ*p*(*l*)) was estimated:

$$\Delta p(l)=p_{y}(l)-p_{x}(l)$$ (4)

The 95% CI’s for Δ*p*(*l*) were obtained based on the two bootstrap population results for *p*_*x*_(*l*) and *p*_*y*_(*l*), respectively. As they are obtained independently of each other, a new bootstrap population of results for Δ*p*(*l*) was created using the methods described in [10]:

$${\Delta p(l)}_{i}={p_{y}(l)}_{i}-{p_{x}(l)}_{i}i\in [1\ldots 1000]$$ (5)

Based on this final bootstrap population, Efron 95% percentile CI’s were obtained for Δ*p*(*l*) as described above.

### 2.4 Predicting the effect of grid bar spacing on gear size selectivity

To investigate the potential for improving species and size selectivity in the shrimp trawl by changing the bar spacing in the Nordmøre grid, the combined size selectivity (Fig 1) *r*_*comb*_(*l*) of a 35 mm codend preceded by different bar spacing Nordmøre grids was estimated.

Using estimates for grid passage probability *p*_*x*_(*l*) where subscript *x* stands for 17, 19 or 21 mm bar distance, we predicted the combined selectivity for each grid and codend configuration. For the 17 and 21 mm bar spacing grids, these estimates were obtained from this study, while for the 19 mm bar spacing grid, results from [10] were used. For codend size selection *r*_*codend*_(*l*) the estimates used for the standard 35 mm diamond mesh codend were obtained from [10, 36]. Specifically, using these results the combined selectivity was predicted by:

$$r_{comb}(l)=p_{x}(l)\times r_{codend}(l)$$ (6)

This prediction approach is similar to the one applied by [37]. Uncertainties in terms of 95% percentile CI’s for *r*_*comb*_(*l*) were obtained based on the individual bootstrap population for *p*_*x*_(*l*) and *r*_*codend*_(*l*) previously used to estimate uncertainties for these curves individually. Thus, we estimated the uncertainty for a dual sequential process based on bootstrap populations used to estimate the uncertainty estimation of the individual processes [36]:

$${r_{comb}(l)}_{i}={p_{x}(l)}_{i}\times {r_{codend}(l)}_{i}\;i\in [1\ldots 1000],$$ (7)

where *i* denotes the bootstrap repetition index. As resampling was random and independent for both groups of results, it is valid to generate the bootstrap population of results for the product based on (7) using two independently generated bootstrap files [36]. The difference in *r*_*comb*_(*l*) for using different Nordmøre grid bar spacings was estimated from the two bootstrap populations using the approach described in Eq (5).

### 2.5 Predicting the effect of grid bar spacing on catch size distribution in the shrimp trawl

To investigate how the application of different Nordmøre grid bar spacings would affect the catch size distribution in the Northern shrimp fishery, we estimated the size-dependent fraction *nrPop*_*l*_ of the populations of shrimp and bycatch species that would be retained in the trawl by:

$${nrPop}_{l}=r_{comb}(l)\times {nPop}_{l},$$ (8)

where *nPop*_*l*_ is the population entering the trawl in front of the Nordmøre grid section in terms of number of individuals. For this population, we used the sum for all hauls in this study (i.e., those obtained with the 17 and 21 mm Nordmøre grid bar spacings). Parameter *n* is the number of individuals in length class *l*. Uncertainties in terms of 95% percentile CI’s for *nrPop*_*l*_ were obtained based on the individual bootstrap population for *r*_*comb*_(*l*) and for *nPop*_*l*_ following the approach in [38].

The value of a catch efficiency indicator *nP+* was estimated for Northern shrimp and bycatch species. This indicator is often used in fishing gear size selectivity studies to supplement selectivity curve assessment [37, 39–42]. It quantifies the retention efficiency of the catch above a specified length *L* for the population entering the fishing gear [43]. Ideally *nP+* should be high (close to 100) for the target species and low for the bycatch species (close to 0). Parameter *nP+* was estimated by:

$$nP+=100\times \frac{\sum_{l>L}{nrPop}_{l}}{\sum_{l>L}{nPop}_{l}}$$ (9)

For the Northern shrimp, Eq (9) was used with CLs *L* set at 15 mm (for industrial shrimp above the MLS) and 20 mm (for commercial size individuals of higher value), respectively. For bycatch species, Eq (9) was used summed over all lengths of measured fish (up to 40 cm length).

Further, we quantified the fraction of the catch which consists of undersized Northern shrimp retained by grids with 17, 19 and 21 mm bar spacings, respectively (*nDiscardRatio*). The *nDiscardRatio* is estimated as follows:

$$nDiscardRatio=100\times \frac{\sum_{i=1}^{h}\sum_{l=0}^{MLS}n_{il}}{\sum_{i=1}^{h}\sum_{l}n_{il}},$$ (10)

where *h* equals number of hauls and *i* is the specific haul with 17 mm, 19 and 21 mm grid, respectively. Parameter *l* is the CL class and MLS is the minimum landing size of shrimp corresponding to 15 mm CL. The discard ratios are given in percentage and the values should ideally be as low as possible.

## 3. Results

### 3.1. Grid passage probability

#### 3.1.1. Sea trials and collected data

The sampling effort and catch data for Northern shrimp, cod, and American plaice is presented in Table 1 (S1 and S2 Files).

**Table 1:** Overview of the hauls carried out and number (*n*) of individuals measured in each haul during the sea trials.

Grid	Haul	Towing time (min)	Depth (m)	*n* shrimp		*n* cod		*n* American plaice	
				*C*	*GC*	*C*	*GC*	*C*	*GC*
17 mm	1	70	241	172 (0.110)	0 (1.000)	39	96	14	53
17 mm	2	76	259	202 (0.073)	63 (1.000)	16	43	33	113
17 mm	3	64	253	188 (0.095)	43 (0.890)	24	69	24	137
17 mm	4	42	257	188 (0.087)	17 (1.000)	76	250	35	35
17 mm	5	61	198	246 (0.020)	224 (0.445)	72	338	32	191
17 mm	6	61	173	236 (0.012)	234 (0.260)	140	713	52	259
17 mm	7	60	195	290 (0.011)	177 (0.394)	94	795	125	399
17 mm	8	54	173	340 (0.023)	189 (0.489)	69	926	113	274
17 mm	9	59	193	304 (0.015)	205 (0.488)	142	1349	97	346
17 mm	10	61	197	277 (0.071)	215 (0.347)	132	827	67	174
17 mm	11	56	247	185 (0.052)	42 (0.922)	155	67	98	57
21 mm	12	60	201	323 (0.432)	187 (1.000)	502	1704	108	267
21 mm	13	59	173	240 (0.020)	159 (0.884)	689	1990	150	201
21 mm	14	60	183	266 (0.029)	93 (0.726)	802	2845	83	209
21 mm	15	61	215	270 (0.021)	57 (0.978)	414	1112	65	108
21 mm	16	58	206	229 (0.032)	181 (0.769)	1180	2981	61	203
21 mm	17	56	251	185 (0.112)	26 (0.945)	43	105	14	46
21 mm	18	56	255	219 (0.039)	53 (0.632)	108	153	25	81
21 mm	19	58	236	212 (0.041)	140 (0.35)	111	273	24	124
21 mm	20	61	254	202 (0.021)	122 (0.938)	97	274	22	57
21 mm	21	56	245	185 (0.040)	71 (0.916)	136	327	21	43
21 mm	22	60	253	184 (0.066)	65 (0.846)	157	236	10	37
21 mm	23	62	271	160 (0.135)	3 (1.000)	28	21	12	15
21 mm	24	60	224	183 (0.083)	15 (0.938)	129	50	44	42
21 mm	25	60	186	168 (0.113)	14 (0.840)	79	5	67	25
21 mm	26	58	251	200 (0.058)	30 (0.783)	150	37	140	73
21 mm	27	60	229	153 (0.075)	16 (1.000)	23	87	88	80
21 mm	28	62	273	170 (0.120)	3 (1.000)	16	102	27	119
21 mm	29	56	277	225 (0.080)	18 (0.812)	49	60	19	58
21 mm	30	60	252	156 (0.068)	18 (0.718)	55	148	35	55
21 mm	31	56	252	205 (0.066)	36 (1.000)	298	72	63	55
21 mm	32	60	232	160 (0.086)	4 (0.724)	32	89	70	84

*C* = codend; *GC* = grid cover. Numbers in parentheses are subsampling factors. In some cases shrimps from the *GC* sub-sample were not possible to length-measure due to broken carapace.

#### 3.1.2. Northern shrimp

**Fig 2:**
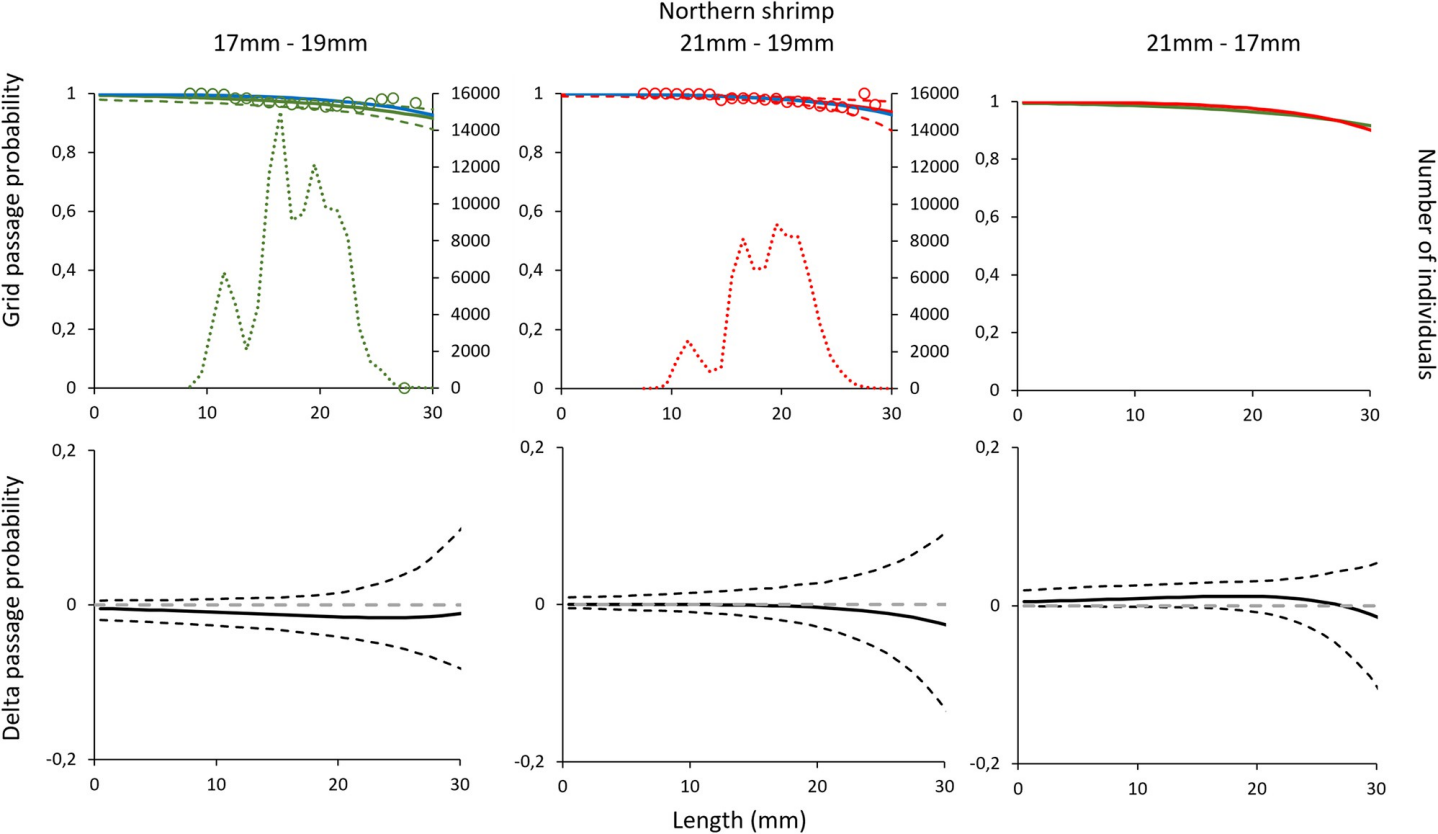
Grid passage probability for Northern shrimp (a-c). Circles illustrate experimental rates, solid curves represent the fitted model, and stippled curves are the 95% CI’s. Green lines and circles represent the data for grid with 17 mm bar spacing, red– 21 mm grid spacing, and blue line–grid with 19 mm grid spacing. Dotted green and red lines represent total population size structure retained by the gear. Plots d-f show the difference in the length-dependent grid passage probability (delta) between the different grids with 95% CI’s (stippled lines).

Comparing the grid passage probability for Northern shrimp demonstrated no significant difference between the grids with three bar spacings (i.e., 17 mm vs 19 mm, 21 mm vs 19 mm and 21 mm vs 17 mm) (Fig 2). The contact probability for Northern shrimp was nearly 100% for all three grids (Table 2), meaning that very few shrimp are released through the escape outlet without contacting the grid. The high *L50*_*grid*_ and *SR*_*grid*_ values estimated are caused by the extrapolation from the model and are biologically meaningless considering the size range of Northern shrimp. Therefore, they should be considered only as parameters necessary for model estimation. The model used to represent the experimental data for shrimp fitted well. Thus, the low values obtained were most likely a consequence of over-dispersion in the experimental catch portioning data that resulted from working with pooled and subsampled data with low sampling rates (Table 2).

**Table 2:** Selectivity parameters and fit statistics for Northern shrimp describing the grid passage probability for the three different grids considered in this study.

	17 mm	19 mm*	21 mm
*C*_*grid*_	1.00 (0.99–1.00)	1.00 (0.97–1.00)	1.00 (0.99–1.00)
*L50*_*grid*_	56.30 (45.96–94.87)	48.87 (28.07–197.42)	52.13 (40.45–88.83)
*SR*_*grid*_	24.16 (16.40–51.66)	16.45 (0.10–41.74)	18.05 (11.27–37.32)
*p*-value	< 0.001	0.097	< 0.001
**Deviance**	160.26	23.65	59.39
**DOF**	19	16	20

95% CI’s are shown in parentheses. DOF = degree of freedom.

*Results from Larsen et al. [10].

#### 3.1.3. Cod

**Fig 3:**
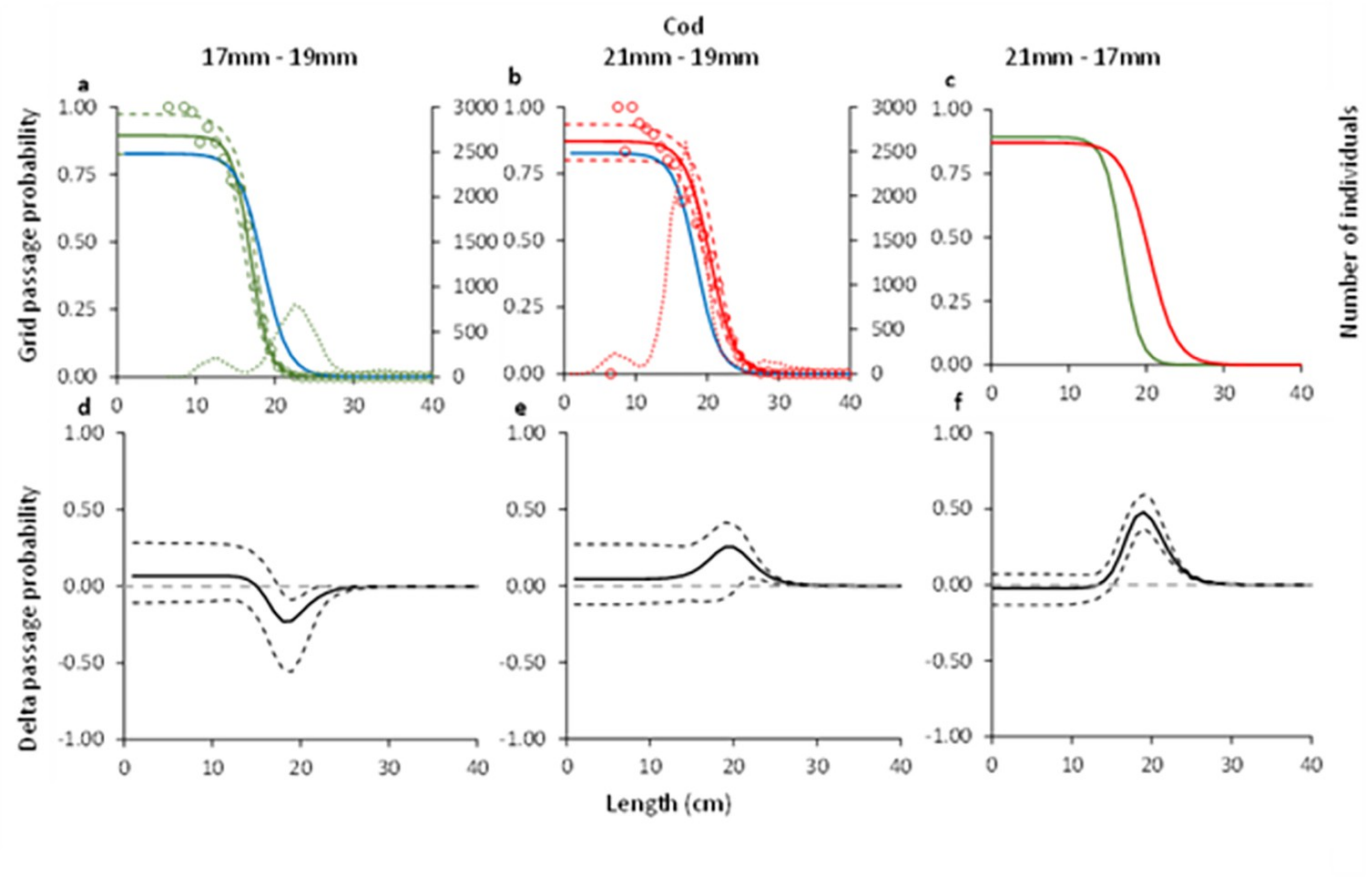
Grid passage probability for cod (a-c). Circles illustrate experimental rates, solid curves represent the fitted model, and stippled curves are the 95% CI’s. Green lines and circles represent the data for grid with 17 mm bar spacing, red– 21 mm grid spacing, and blue line–grid with 19 mm grid spacing. Dotted green and red lines represent total population size structure retained by the gear. Plots d-f show the difference in the length-dependent grid passage probability (delta) between the different grids with 95% CI’s (stippled lines).

Comparisons of the grid passage probability for cod through the three grid bar spacings demonstrated significant differences between them, i.e., 17 mm vs 19 mm, 21 mm vs 19 mm and 21 mm vs. 17 mm (Fig 3). The delta plots illustrate that the difference between the grid passage probability is largest between the 17 mm and 21 mm bar spacing grids for cod (Fig 3F).

The grid contact probability estimated for cod with the 17 and 21 mm grids was similar and did not differ significantly from the value for *C*_*grid*_ estimated by Larsen et al. [10] (Table 3). The *L50*_*grid*_ of 20.4 cm for the 21 mm was significantly higher than for the 17 mm which was 16.9 cm. The *L50*_*grid*_ for the 19 mm grid estimated by Larsen et al. [10] was 18.55 (CI: 15.93–21.46) cm, which is in between the values for the 17 and 21 mm grids (Table 3).

**Table 3:** Selectivity parameters and fit statistics for cod describing the grid passage probability for the three different grids considered in this study.

	17 mm	19 mm*	21 mm
*C*_*grid*_	0.89 (0.82–0.97)	0.83 (0.70–1.00)	0.87 (0.80–0.93)
*L50*_*grid*_	16.91 (16.12–17.53)	18.55 (15.93–21.46)	20.40 (19.49–21.32)
*SR*_*grid*_	2.53 (2.11–3.00)	5.06 (1.15–7.31)	3.77 (3.04–4.50)
*p*-value	0.6527	0.9976	< 0.001
**Deviance**	27.38	15.98	111.34
**DOF**	31	35	32

The 95% CI’s are shown in parentheses. DOF = degree of freedom.

*Results from Larsen et al. [10].

#### 3.1.4. American plaice

**Fig 4:**
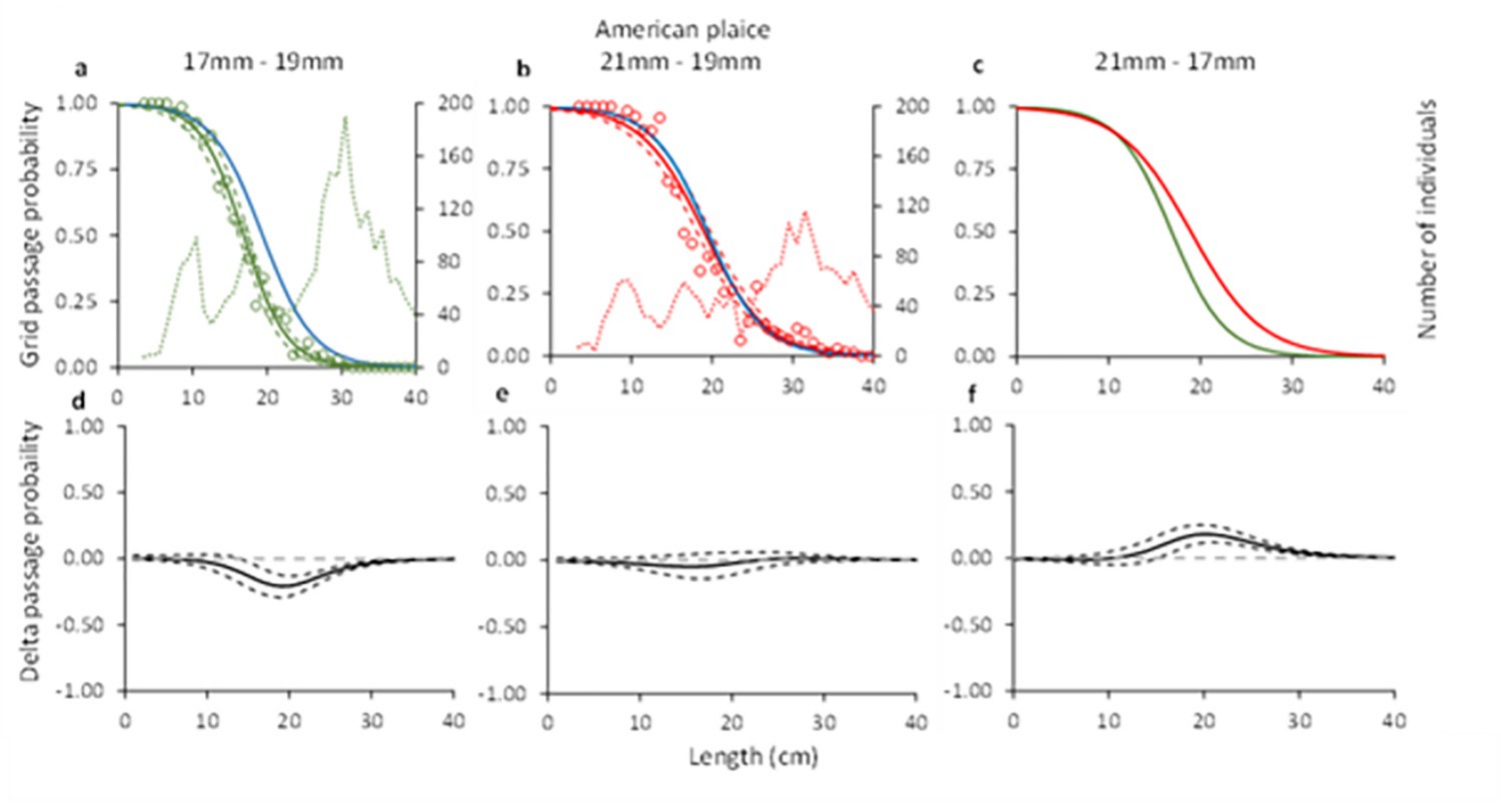
Grid passage probability for American plaice (a-c). Circles illustrate experimental rates, solid curves represent the fitted model, and the stippled curves are the 95% CI’s. Green lines and circles represent the data for grid with 17 mm bar spacing, red– 21 mm grid spacing, and blue line–grid with 19 mm grid spacing. Dotted green and red lines represent total population size structure retained by the gear. Plots d-f show the difference in the length-dependent grid passage probability (delta) between the different grids with 95% CI’s (stippled lines).

Comparing the grid passage probability for American plaice for the 17, 19 and 21 mm grids demonstrated significant differences in two out of the three comparisons (e.g., 17 mm vs 19 mm and 21 mm vs 17 mm). The delta plot for the grid passage probability between the 19 and the 21 mm grids showed no significant differences (Fig 4). The *C*_*grid*_ value estimated for the three grids was 100% in all three cases (Table 4). The *L50*_*grid*_ estimated for the 21 mm grid was 18.9 cm and differed significantly from that estimated for the 17 mm grid, which was 16.8 cm (Table 4). The *L50*_*grid*_ value for the 19 mm differed significantly from the value estimated for the 17 mm grid but did not differ from that estimated for the 21 mm grid (Table 4).

**Table 4:** Selectivity parameters and fit statistics for American plaice describing the grid passage probability for the three different grids considered in this study.

	17 mm	19 mm *	21 mm
*C*_*grid*_	1.00 (1.00–1.00)	1.00 (0.96–1.00)	1.00 (1.00–1.00)
*L50*_*grid*_	16.85 (16.18–17.56)	19.46 (18.29–20.98)	18.94 (18.10–19.74)
*SR*_*grid*_	6.34 (5.33–7.35)	8.26 (6.68–9.53)	8.45 (7.69–9.29)
*p*-value	0.9003	0.9524	0.0533
**Deviance**	31.61	25.54	61.29
**DOF**	43	39	45

The 95% CI’s are shown in parentheses. DOF = degree of freedom.

* Results from Larsen et al. [10].

### 3.2. Effect of grid bar spacing on gear size selectivity and catch size distributions

#### 3.2.1. Northern shrimp

**Fig 5:**
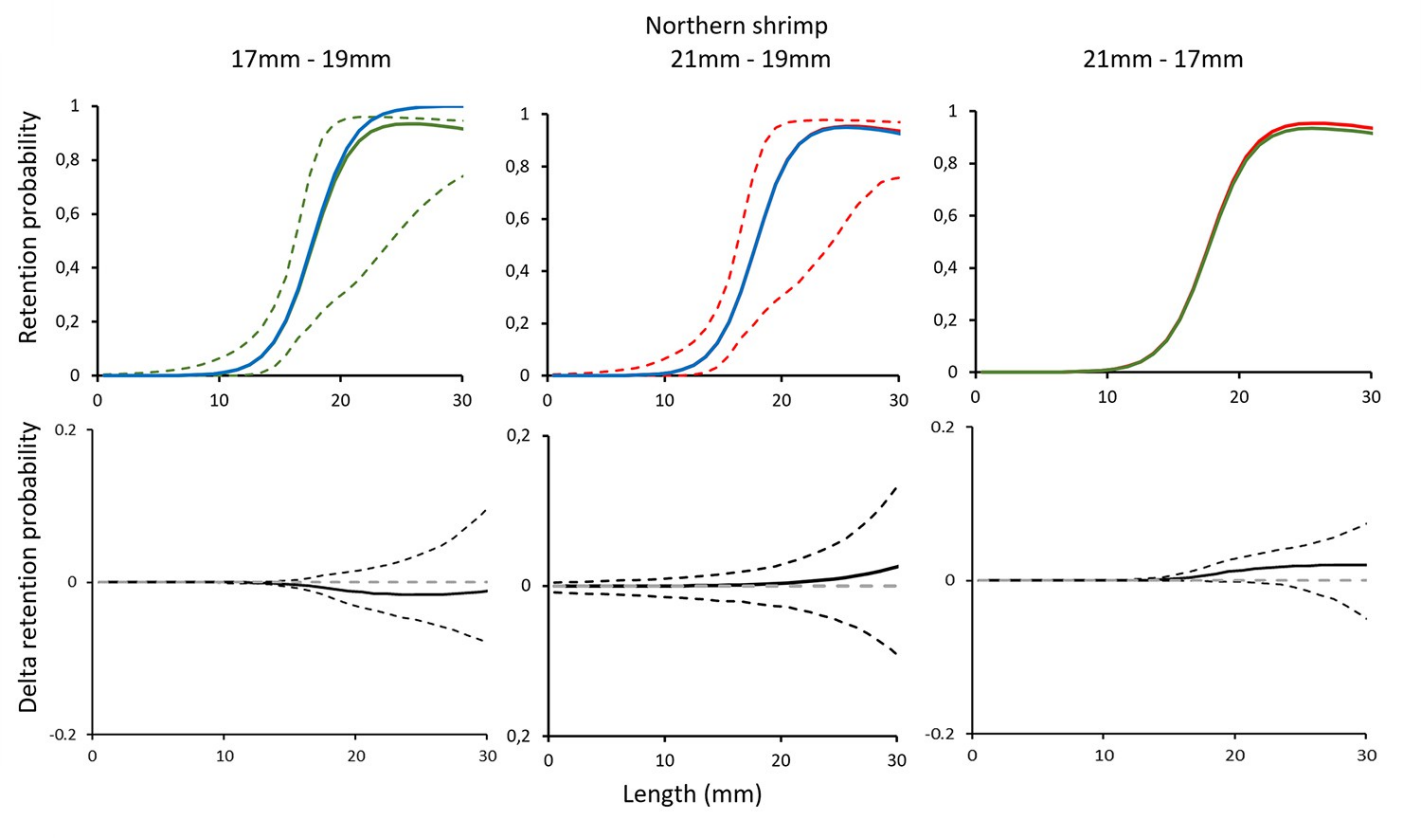
Combined (grid and codend) selectivity curves (a-c) and delta plots (d-f) obtained with different grid bar spacing for Northern shrimp. In plots a-c, the green curve shows results for 17 mm, blue– 19 mm, and red– 21 mm grid spacing. Stippled lines are the 95% CI’s.

**Fig 6:**
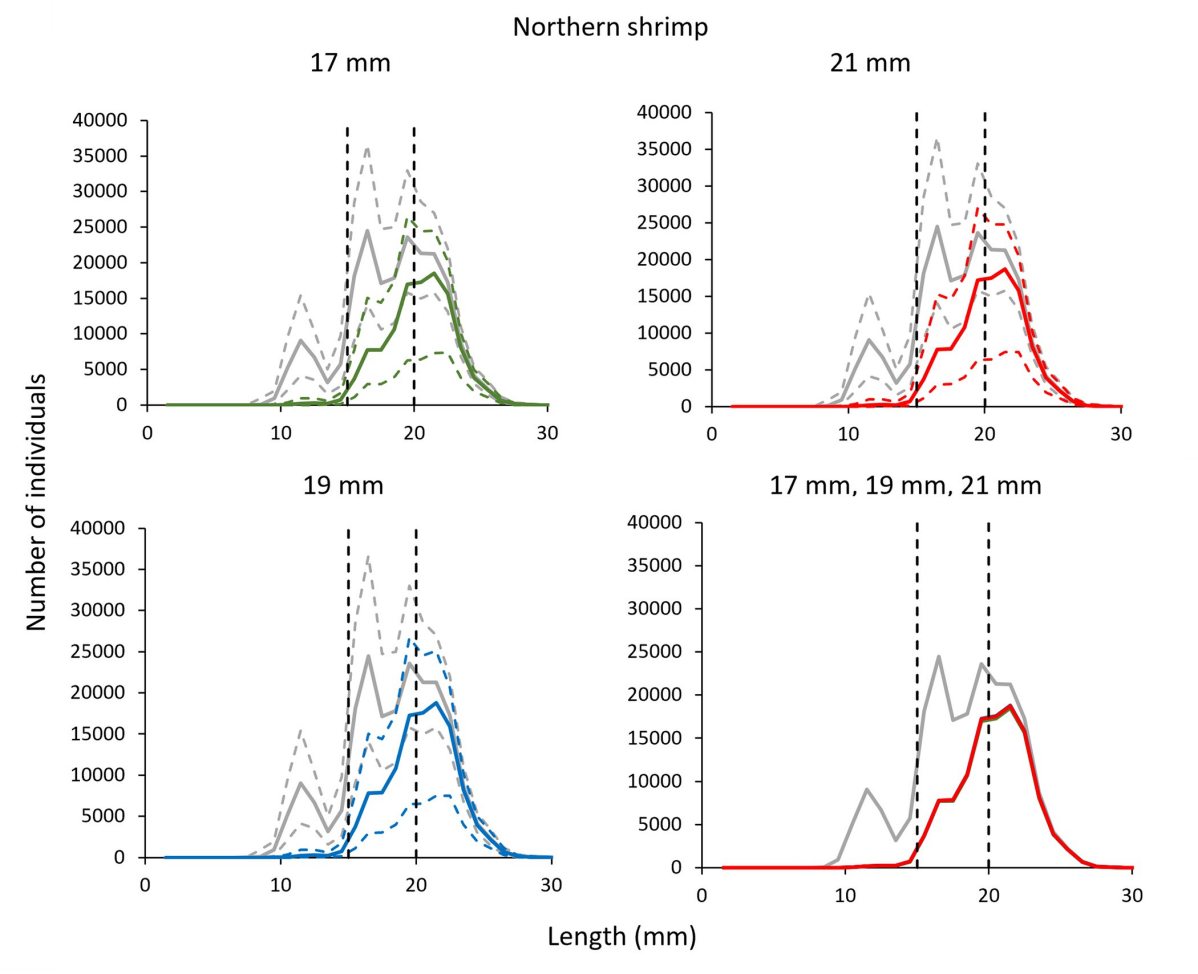
Number of retained Northern shrimp and the entry population (grey line) when using grid with 17 mm (green), 19 mm (blue) and 21 mm (red) bar spacing. Stippled lines represent the 95% CI’s.

The predicted retention probability curves for the combined effect of the grid and codend demonstrated no significant difference between the three grids (i.e., bar spacings 17 mm vs 19 mm, 21 mm vs 19 mm, and 21 mm vs 17 mm grids combined with a 35 mm codend) (delta plots in Fig 5). This is also corroborated by the length distributions retained by the three gear configurations with entry populations of shrimp, which were nearly identical (Fig 6).

The catch efficiency indicators (*nP+*) show that the capture probability for both shrimp > 15 mm CL (i.e., larger than the MLS [11]) and shrimp > 20 mm CL was only slightly different between the different bar spacings considering the size structure of the population caught. These differences were not significant between any of the three grids (Table 5). Changing the grid bar spacing from 19 mm to 17 mm only decreased the retention probability by 1.56% for shrimp > 15 mm CL, and by 1.64% for shrimp > 20 mm CL. On the other hand, increasing the grid bar spacing from 19 mm to 21 mm only increased the retention probability for shrimp > 15 mm CL by 0.10%, and 0.16% for shrimp > 20 mm CL (Table 6). Further, the discard ratio which here is the estimated proportion of shrimp below the MLS in the population encountered, was not significantly different for the three grids considered in the study (Table 5).

**Table 5:** Catch efficiency indicators *nP+* (in %) for Northern shrimp with carapace lengths >15 mm and >20 mm, respectively, and bycatch species (cod and American plaice) and fraction of undersized shrimp (*nDiscardRatio*) (in %) when using the 17, 19 and 21 mm bar spacing grids combined with the 35 mm codend for the population encountered.

	*nP+* (%)				*nDiscardRatio* (%)
Grid	Shrimp >15 mm	Shrimp >20 mm	Cod	American plaice	Shrimp
17 mm	63.90 (29.95–80.09)	87.43 (38.14–95.38)	6.03 (3.00–10.76)	12.83 (10.21–15.31)	1.28 (0.22–4.71)
19 mm	64.91 (30.86–79.50)	88.88 (39.61–96.88)	12.84 (8.66–16.91)	19.09 (15.98–22.29)	1.27 (0.22–4.69)
21 mm	64.98 (30.05–81.31)	89.02 (38.99–97.27)	25.15 (12.83–31.51)	19.13 (16.02–22.27)	1.27 (0.22–4.67)

**Table 6:** Change in the catch efficiency indicator *nP+* (in %) for Northern shrimp with carapace lengths >15 mm and >20 mm, respectively and bycatch species (cod and American plaice) and fraction of undersized shrimp (*nDiscardRatio*) (in %) when using the 17 and 21 mm bar spacing with respect to the 19 mm bar spacing (baseline) for the population encountered.

	Change in *nP+* (%)				Change in *nDiscardRatio* (%)
Grid	Shrimp >15 mm	Shrimp >20 mm	Cod	American plaice	Shrimp
17 mm	-1.56 (-4.37–2.04)	-1.64 (-4.73–2.73)	-53.06 (-73.51 –-14.41)	-32.76 (-41.97 –-22.71)	0.43 (-1.67–1.97)
21 mm	0.10 (-2.42–3.84)	0.16 (-2.93–4.57)	95.85 (7.04–193.51)	0.24 (-13.29–15.70)	-0.12 (-2.17–1.57)

#### 3.2.2. Cod

**Fig 7:**
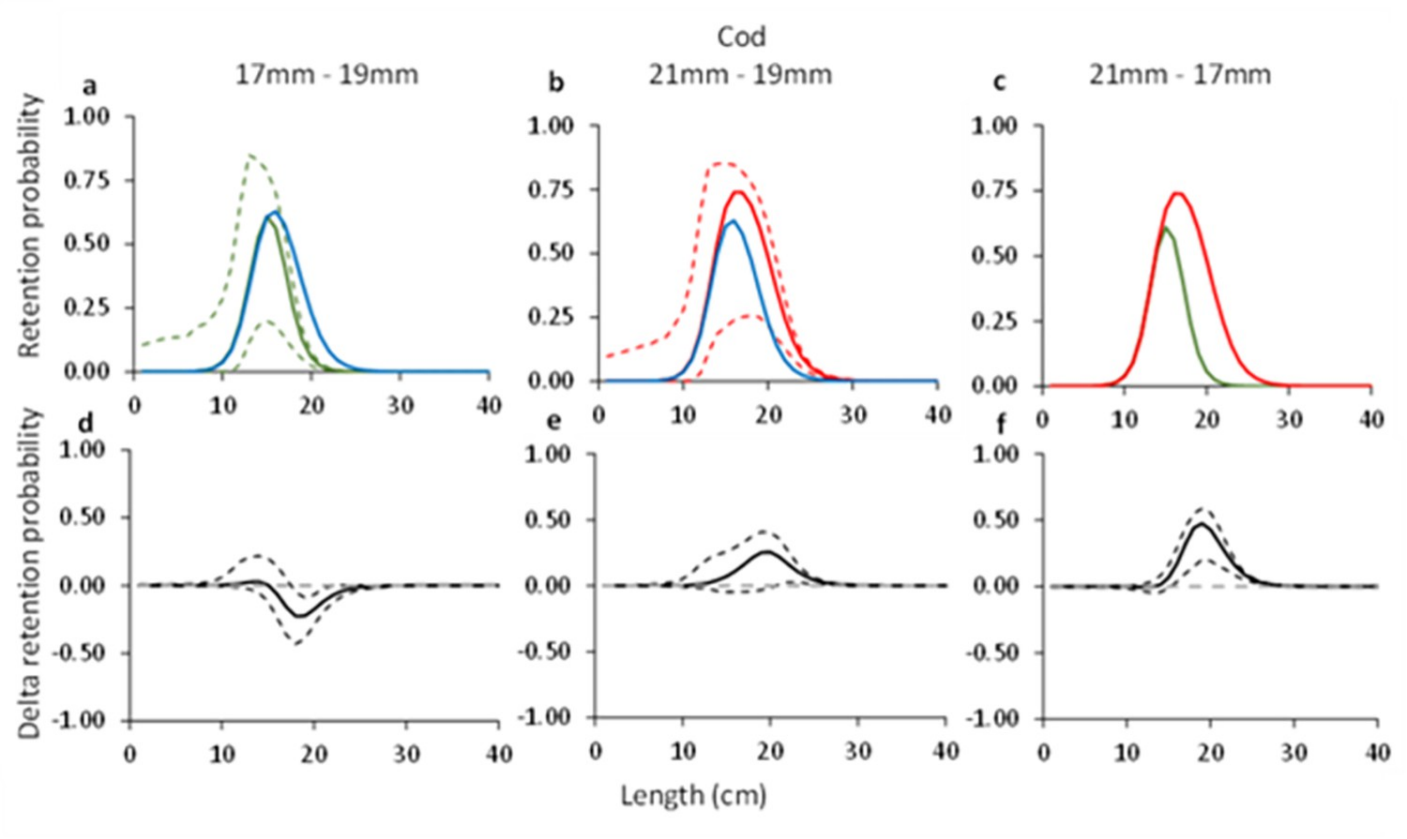
Combined (grid and codend) selectivity curves (a-c) and delta plots (d-f) obtained with different grid bar spacing for cod. In plots a-c, the green curve shows results for 17 mm, blue– 19 mm, and red– 21 mm grid spacing. Stippled lines are the 95% CI’s.

**Fig 8:**
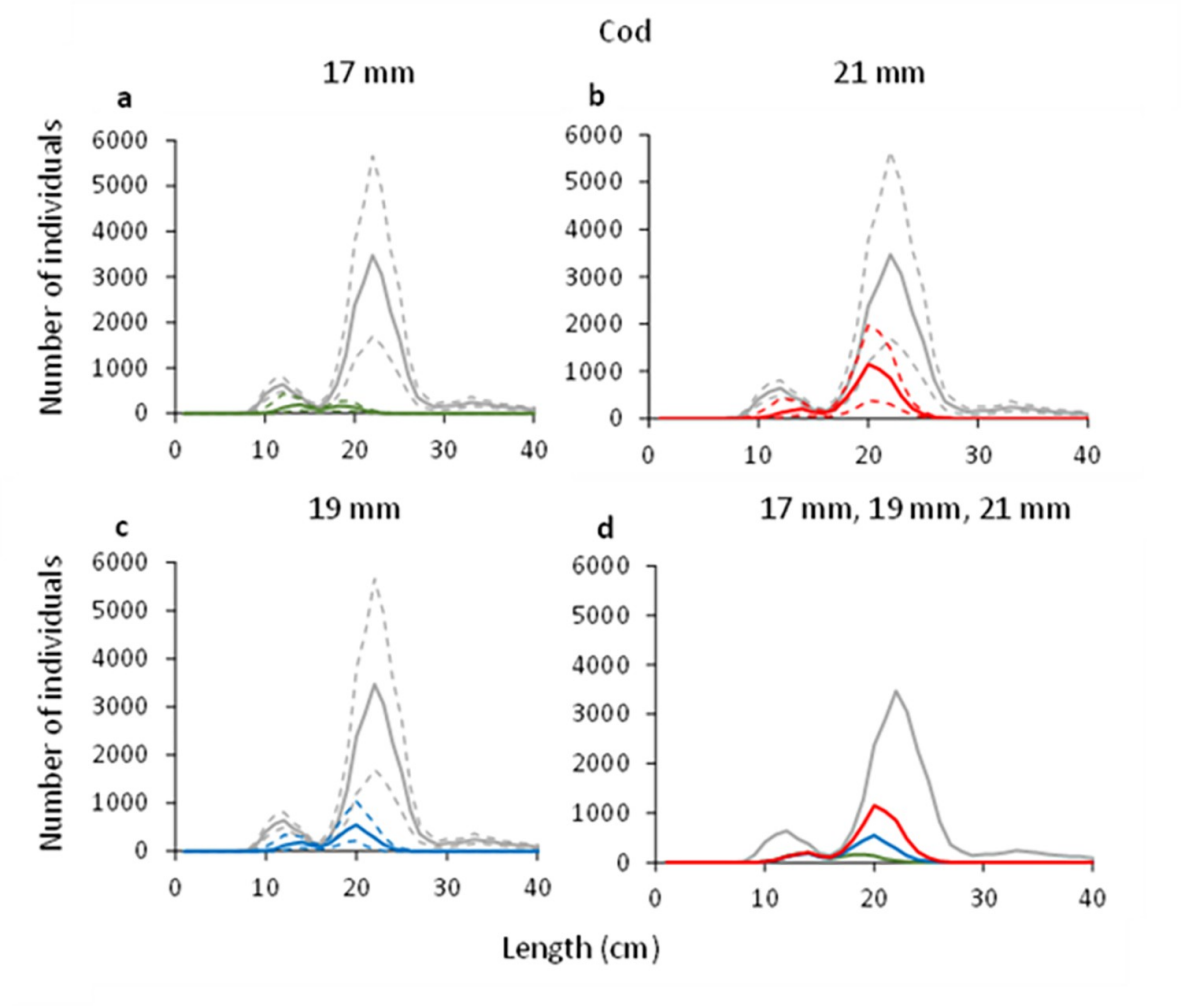
Number of retained cod and the entry population (grey line) when using grid with 17 mm (green), 19 mm (blue) and 21 mm (red) bar spacing. Stippled lines represent the 95% CI’s.

For cod, the predicted retention probability curves for the combined effect of the grid and codend demonstrated a significant difference between the three different grid and codend configurations (e.g., 17 mm vs 19 mm, 21 mm vs 19 mm, and 21 mm vs 17 mm grids combined with a 35 mm codend) (Fig 7). The configuration with the 17 mm bar spacing retained significantly less cod than the configuration with 19 mm bar spacing in the grid. The configuration with the 21 mm bar spacing grid on the other hand retained significantly more cod compared to the configuration with the 19 mm bar spacing grid, a significance that was even more prominent when compared to this configuration with the 17 mm bar spacing (Fig 7). This is also corroborated by the length distributions retained by the three different configurations, which show an increased retention for cod in the configurations with smaller bar spacing (Fig 8). The estimated catch efficiency indicator (*nP+*) showed that the catches of cod doubles when the grid bar spacing is increased from 17 mm to 19 mm, and again when the bar spacing is increased from 19 mm to 21 mm. The difference in catches is significant between the 17 mm grid and the 21 mm grid considering the size structure of the population caught (Table 5). The results also show a significant decrease in the retention probability for cod of 53.06% when grid bar spacing is decreased from 19 mm to 17 mm, and contrary, a significant increase of 95.85% when grid bar spacing is increased from 19 to 21 mm (Table 6).

#### 3.2.3. American plaice

**Fig 9:**
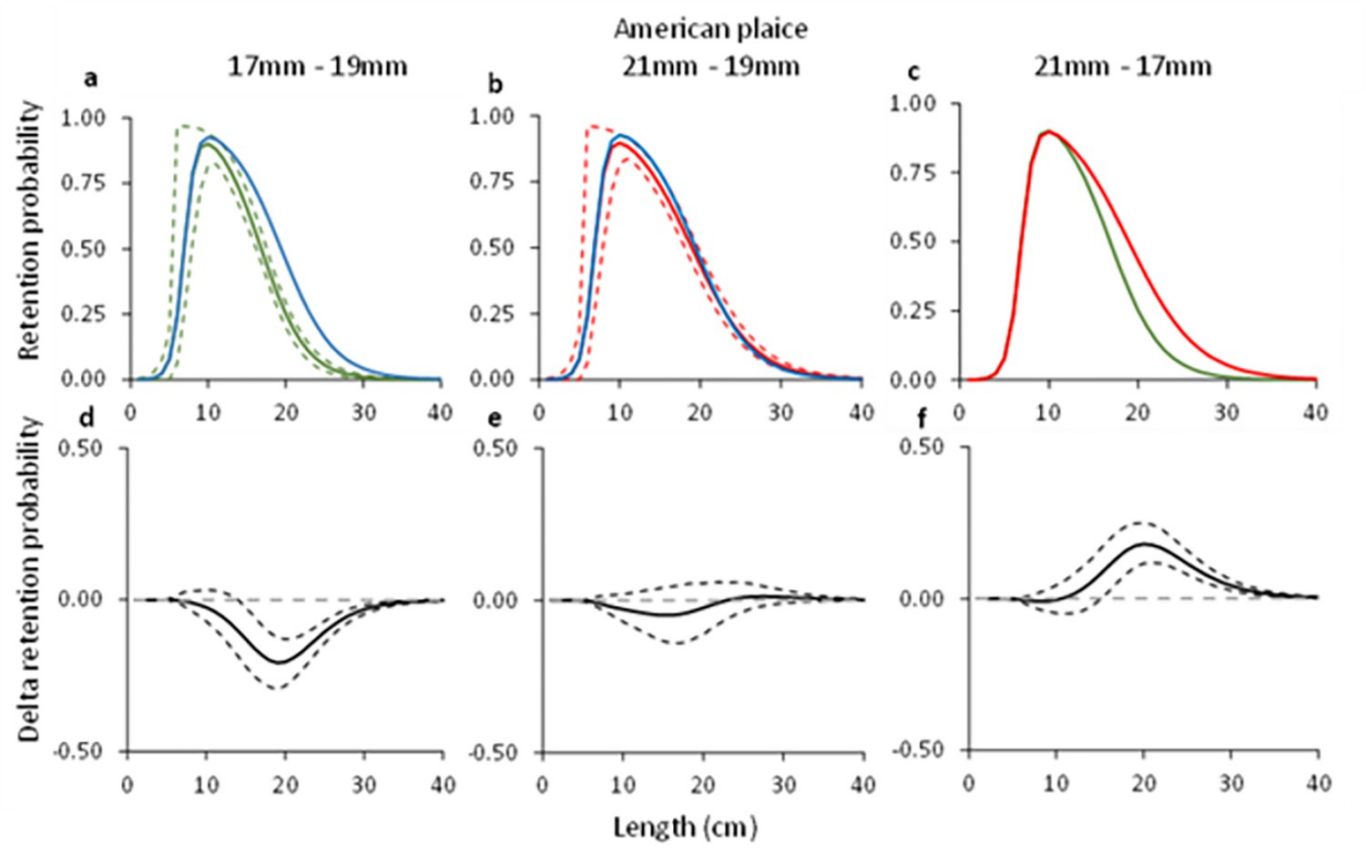
Combined (grid and codend) selectivity curves (a-c) and delta plots (d-f) obtained with different grid bar spacing for American plaice. In plots a-c, the green curve shows results for 17 mm, blue– 19 mm, and red– 21 mm grid spacing. Stippled lines are the 95% CI’s.

**Fig 10:**
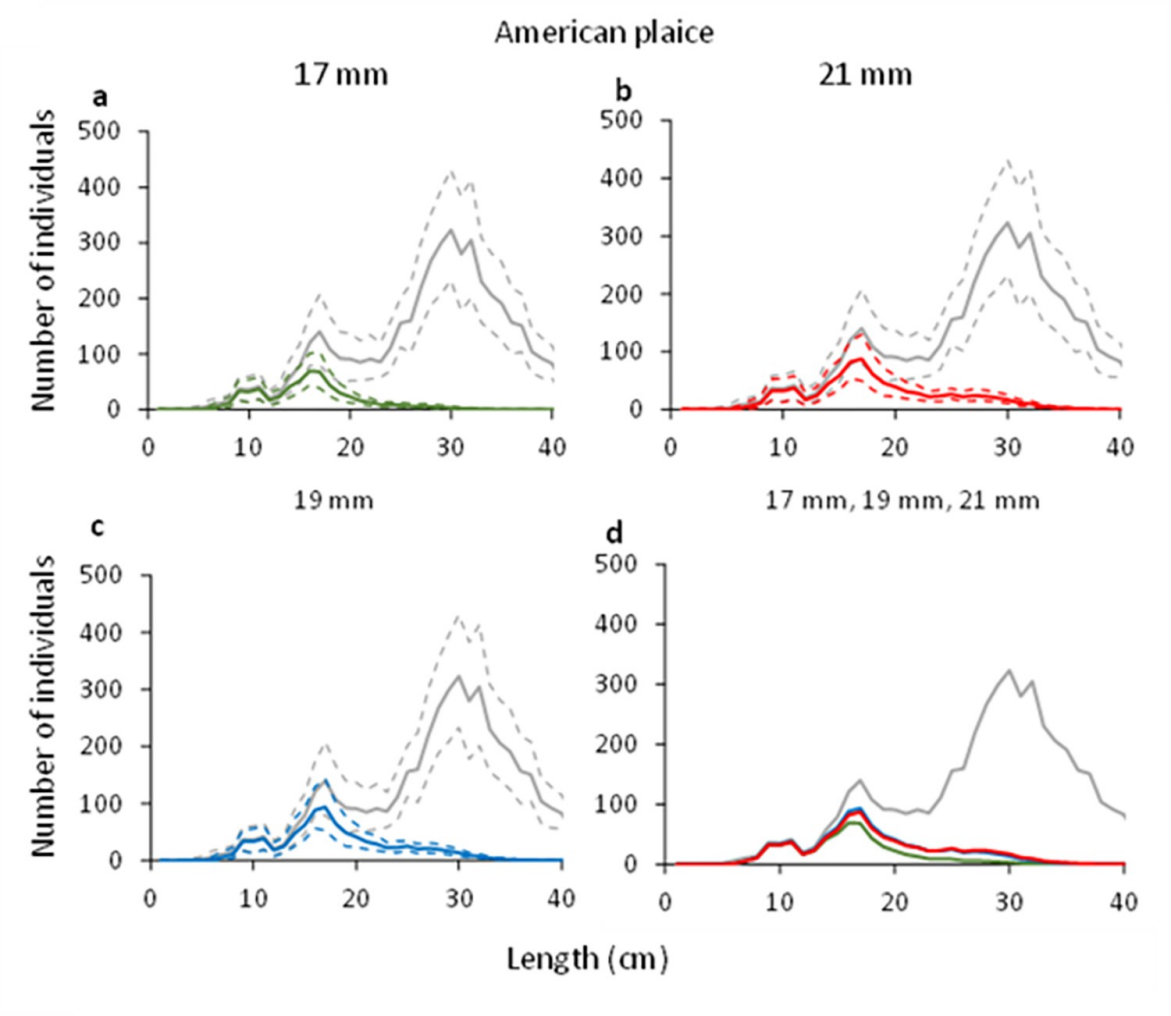
Number of retained American plaice and the entry population (grey line) when using grid with 17 mm (green), 19 mm (blue) and 21 mm (red) bar spacing. Stippled lines represent the 95% CI’s.

For American plaice, the predicted retention probability curves for the combined effect of the grid and codend demonstrated a significant difference between the configuration with the 17 mm grid and the 19 mm grid, and the configurations with the 21 mm grid and 17 mm grid (Fig 9). No difference was observed between the configurations with the 21 mm and the 19 mm grid (Fig 9). The configuration with the 17 mm bar spacing grid retained significantly less American plaice than that with the 19 mm bar spacing grid (Fig 9). The configuration with the 21 mm bar spacing grid on the other hand, retained significantly more American plaice compared to the configuration with 17 mm bar spacing grid (Fig 9). This is also corroborated by the length distributions retained, which show a similar length distribution for the configurations with the 19 mm and 21 mm grids, whereas the retention for the configuration with the 17 mm bar spacing grid was lower (Fig 10). The catch efficiency indicator (*nP*+) for American plaice shows that the catches significantly increase when grid bar spacing is increased from 17 mm to 19 mm, while it barely changes when the bar spacing is increased from 19 to 21 mm considering the size structure of the population caught (Table 5). The differences in catches are also reflected in the change in capture probability for American plaice, which significantly decreases by 32.76% when decreasing grid bar spacing from 19 mm to 17 mm, but barely increases when increasing grid bar spacing from 19 mm to 21 mm (Table 6).

## 4. Discussion

In the Barents Sea Northern shrimp trawl fishery, the use of a Nordmøre grid with 19 mm bar spacing to supplement codend size selectivity is mandatory to reduce bycatch. In the present study, we investigated the effect of changing grid bar spacing on the size selectivity of Northern shrimp and two bycatch species: cod, and American plaice.

The results obtained for Northern shrimp show that increasing grid bar spacing from the compulsory 19 mm to 21 mm or, on the contrary, reducing it to 17 mm did not have significant consequences regarding the passage probability of shrimp (Fig 2D–2F). Further, the retention probability curves for the combined effect of the grid and codend did not show significant differences between the three different grid and codend configurations considered (Fig 5D–5F). Northern shrimp have limited swimming capability and, therefore, they are able to pass through the grid if they contact the device with optimal orientation [23]. The results of this study show that the grid passage probability estimated for shrimp is close to 100% for the 17, 19 and 21 mm grid (Table 2). However, the results presented in this study need to be interpreted correctly considering the population size structure, especially regarding grid passage probability since it is dependent on the size structure of the shrimp population entering the trawl.

The recorded sizes of the Northern shrimp reaches up to 32 mm [44]. If shrimp size structure in a fishing ground were larger compared to the size structure in this study and if the shrimp were present in sufficient numbers, the portion of the curve with lower passage probabilities could be visible. This would most likely show differences between the grids with different bar spacings. Therefore, the results obtained for shrimp grid passage probability in this study should be interpreted keeping in mind that in areas with larger shrimp than those caught in our study, there could potentially be differences in passage probability for the largest individuals. However, the size structure of Northern shrimp and the bycatch composition obtained in this study is similar to those obtained in several earlier experiments that have been carried out in the Barents Sea during the last two decades [10, 16, 21–25, 45]. This shows that there is likely little variation in the shrimp population size structure in the Barents Sea. Therefore, it makes the results presented here applicable for the whole area and the comparisons carried out with the 19 mm grid from Larsen et al. [10] appropriate. Further, the data for the 19 mm grid were collected under similar environmental conditions and by the same crew, using the same vessel and trawl configuration, and following the same sampling protocols.

Regarding the selectivity results obtained for the two bycatch species, the grid passage probability of both cod and American plaice increase with increased bar spacing. The difference between the grids is largest when the grids with 17 and 21 mm bar spacing are compared (Figs 3D–3F and 4D–4F). When the predicted retention probability curves for the combined grid and codend configurations are compared for the 17, 19 and 21 mm grids, the results show a pattern that is similar to that observed for the differences in grid passage probability alone. Specifically, it shows that the sorting grid has a major role in the overall selection process of the combined gear (Figs 8D–8F and 9D–9F).

The catch efficiency indicator (*nP+*) for the shrimp > 15 mm is ca. 24% lower than for the larger shrimp (>20 mm) for the three grid and codend configurations included in this study. However, the difference is not significant in any case. The difference observed is probably due to the size sorting process in the codend, which becomes more relevant for the smaller sizes of shrimp. The *nP+* and discard ratio values were similar for Northern shrimp >15 mm individuals above the MLS) and >20 mm (commercially important sized individuals) for the selective gear configurations tested, showing that the size distribution of shrimp catches were similar in the three grids. There were significant differences in catch efficiency indicator values for cod and American plaice. For both species, the *nP+* values significantly increased with increased bar spacing. This implies that larger number of fish were retained with respect to the numbers that entered the gear with increased bar spacing.

The results of this study show that with the shrimp populations encountered, changing grid bar spacing from 17 to 21 mm does not result in significant changes in shrimp catch, whereas the bycatch of cod and American plaice changes significantly. Therefore, in areas where cod and American plaice populations are abundant and exhibit a similar size structure as in the current study, changing the compulsory grid of 19 mm to a grid of 17 mm would reduce the catches of cod by half and the catches of American plaice by one third while catching the same amount of shrimp. These results show the importance of considering the population structure in this and other shrimp fisheries at the time the fishing operations are conducted. Further, it shows the relevance and potential impact of flexibility in gear choice and supports the use of more dynamic management systems based on new monitoring systems, which is a growing trend in modern fisheries [46].

Several different gear modifications have been tested to improve the selectivity of shrimp trawls. These include, for example, additional grids or sieve panels [10, 24], modified codend designs regarding codend meshes [16, 22], and use of artificial lights [21]. However, the results obtained during those trials were variable. Therefore, more research is required to determine a design that reduces fish and undersized Northern shrimp bycatch while minimizing the loss of target individuals. Therefore, we designed this study to examine the effect of the grid bar distance. Different bar spacing grids have been tested in other shrimp fisheries around the world (e.g., [18, 26–29, 47, 48]). For example, Hickey et al. [26] tested 22, 25 and 28 mm grids along the fishing grounds of Eastern Canada. The results showed while the different grids were effective at reducing bycatch, there was no significant difference in the mean Northern shrimp length captured with either of the grids. In a recent study from the same area (the shrimp grounds off Newfoundland), similar results, i.e., no difference, was obtained for shrimp retention when comparing 19 and 22 mm bar spacings in a Nordmøre grid [29]. Furthermore, it was recorded that both grids retained more than half of all redfish (*Sebastes* spp.) by numbers. The authors explained the observed behaviour of redfish during escape and retention (mainly fish <15 cm) between the grids as a possible effect of rejected waterflow directed towards the escape opening. As in the present study, this result is likely a consequence of the size distribution of Northern shrimp when the study was carried out. The length distributions presented did not contain shrimp ≥30 mm, which is probably necessary to potentially detect differences between grids with grid bar spacings ≥21 mm.

The technical regulations in Norwegian and Russian sectors of the Barents Sea and other Norwegian shrimp fishing grounds are a trade-off between maximizing the capture of the target species and minimizing retention of small fish. A reduction of bycatch of juveniles is generally believed to have a positive effect on the stock recruitment [13]. The industry can on a voluntary basis use grids with smaller bar spacings than the regulated maximum 19 mm to improve selectivity. However, from a practical point of view, it is rational for the management to have one selective system, e.g., the 19 mm Nordmøre grid combined with a 35 mm codend, covering all areas within Norwegian (and Russian) jurisdiction. However, species and size composition can change between fishing areas and time of the year. Having the flexibility to reduce grid bar spacing from 19 to 17 mm could provide fishermen access to closed areas where the established bycatch limits [11] would else be exceeded.

On the other hand, in areas with large sizes of shrimp and low densities of bycatch species, increasing bar spacing could theoretically be beneficial. However, this scenario might be unlikely in the Barents Sea and could not be corroborated by the results in the present study due to recorded size distribution of shrimp.

The present study provides an insight of the effects of changing grid bar spacing on the size selectivity of Northern shrimp and two common bycatch species in the Barents Sea fishery. However, the results and findings could be applicable to other shrimp fisheries where flexibility in the use of different grid bar spacings may be beneficial to maximize the reduction of unwanted bycatch awhile minimizing the loss of target species.

## Supporting information

S1 File (ZIP)

S2 File (ZIP)
